# *Septin 9* Methylation in Nasopharyngeal Swabs: A Potential Minimally Invasive Biomarker for the Early Detection of Nasopharyngeal Carcinoma

**DOI:** 10.1155/2020/7253531

**Published:** 2020-05-05

**Authors:** Jie-Yu Lyu, Jian-Yong Chen, Xiao-Jun Zhang, Meng-Wen Zhang, Geng-Sheng Yu, Liang Zhang, Zhong Wen

**Affiliations:** ^1^Department of Otorhinolaryngology-Head and Neck Surgery, Zhujiang Hospital, Southern Medical University, Guangzhou 510280, China; ^2^Department of Otolaryngology, Jiangmen Central Hospital, Affiliated Jiangmen Hospital of Sun Yat-sen University, Jiangmen 529030, China; ^3^Department of Clinical Laboratory, Jiangmen Central Hospital, Affiliated Jiangmen Hospital of Sun Yat-sen University, Jiangmen 529030, China; ^4^Department of Oncology, Jiangmen Central Hospital, Affiliated Jiangmen Hospital of Sun Yat-sen University, Jiangmen 529030, China; ^5^Translational Medicine Center, Guangdong Women and Children Hospital, Guangzhou 511400, China

## Abstract

Nasopharyngeal carcinoma (NPC) is highly prevalent in Southeast Asia, and an unfavorable outcome is usually attributed to advanced stage NPC. Current methods for the early diagnosis of NPC have limitations in clinical practice. The aim of this study was to investigate the diagnostic ability of *Septin 9* methylation for NPC. A quantitative methylation-sensitive PCR (qMS-PCR) assay was developed to measure the methylation status and levels of *Septin 9* in nasopharyngeal tissues and paired swabs from patients with NPC, chronic nasopharyngitis, and healthy donors. Methylated *Septin 9* was detected in 92% (23/25) of NPC tissues and 25% (4/16) of nasopharyngitis controls (*p* < 0.05). High-frequency hypermethylation with decreased mRNA expression of *Septin 9* in NPC was also identified. Further, *Septin 9* methylation was identified in 90.5% (19/21) of NPC biopsies and 71.4% (15/21) of paired swabs, indicating a good concordance between the two sample types. In addition, methylated *Septin 9* was found in 16 (72.7%) nasal swabs from 22 NPC patients, 2 of 19 (10.5%) nasopharyngitis, but not in any of the healthy controls (*p* < 0.01). The methylation score in nasal swabs of the NPC group was also significantly higher than that of non-NPC controls (*p* < 0.001). Moreover, receiver operating characteristic (ROC) curve analysis showed an area under the curve (AUC) of 0.882 of *Septin 9* methylation tests to discriminate NPC from non-NPC subjects. Our study demonstrated that frequent methylation of *Septin 9* was present in NPC. Its detection in nasopharyngeal swabs may provide a minimally invasive and informative method for identifying early NPC cases.

## 1. Introduction

Nasopharyngeal carcinoma (NPC) is a highly prevalent malignancy in Southeastern Asia and Southern China. It is also known as “Cantonese cancer” due to its unusually high incidence in Guangdong province, China. According to a recent report, about 60,000 Chinese were newly diagnosed with NPC in 2015 and 34,000 deaths from this disease [1]. Unfortunately, the majority of NPC cases are diagnosed at late stages, which is often associated with an unfavorable outcome and prognosis [2]. Thus, early diagnosis is a critical issue in the clinical setting. Diagnosis of NPC mainly relies on clinical symptoms, imaging,immunological examinations, and histopathology. Usually, nasopharyngeal tissues are taken under endoscopy from patients with suspected NPC and subjected to histological examination. However, it is invasive and multiple sampling may result in bleeding and other complications. More importantly, it usually needs one week to finish the pathological diagnosis.

Epstein–Barr virus (EBV) infection plays important roles in the development of NPC, and EBV-related markers become the most common immunological method for the diagnostics of this disease [3]. However, it has high false-positive/negative results because of the low sensitivity/specificity [4, 5]. Moreover, not every NPC patient suffered from EBV infection. For example, EBV is generally absent in type II NPC [6]. In addition, EBV infection does not necessarily result in NPC. Both EBV infection and environmental factors co-contribute to the carcinogenesis of NPC. Taken together, the EBV detection system has some limitations in the diagnostics of NPC. The development of a new strategy for early diagnosis of NPC is imperative.

Tumorigenesis is a multistep process that requires multiple genetic and epigenetic alterations [7, 8]. Of particular interest, DNA methylation is the most widely studied epigenetic modification and is thought to be a hallmark of many human cancers [9, 10]. Compelling evidence has demonstrated that aberrant DNA methylation is an early molecular event during malignant transformation that precedes cancer incidence [11, 12]. Also, DNA methylation is a biological process that integrates external stimuli and host responses. Thus, methylation markers are more suitable for tumor diagnostics [13, 14]. Prior studies have identified some genes hypermethylated in NPC [15–17]; however, those targets may not be applicable to clinical practice due to their relatively low sensitivity and specificity.

Septin 9, also known as MSF1, is a member of the septin family involved in many cellular processes such as cell cycle control, cytokinesis, and vesicle trafficking. The accumulating evidence for its involvement in human neoplasia indicates that *Septin 9* may belong to the class of cancer critical genes [18]. Hypermethylation of *Septin 9* was initially described in colorectal cancer but not in normal tissue, which serves as a noninvasive screening test in plasma for colorectal cancer [19, 20]. Further, *Septin 9* methylation was identified in breast and lung cancer [21, 22]. Our previous project also confirmed that methylated *Septin 9* gradually increased from cervical benign lesions, low/high-grade squamous intraepithelial lesions to cervical cancer (manuscript being submitted). Because it has been shown to be a possible tumor suppressor in human cancers such as ovarian cancer [23], we hypothesized that *Septin 9* may also function importantly in the tumorigenesis of NPC. Thus, it would be interesting to know whether *Septin 9* methylation can be found in NPC, which might be a novel marker for aiding in the early detection of NPC.

In the present study, we determined the methylation status of *Septin 9* in nasopharyngeal tissues and corresponding swabs from patients with NPC and chronic nasopharyngitis as well as healthy controls. Our results demonstrated that high frequency of *Septin 9* methylation was present in NPC and its detection in nasopharyngeal swabs may be a minimally invasive tool for diagnosing NPC.

## 2. Materials and Methods

### 2.1. Patients and Clinical Specimens

The study protocol was approved by the Ethics Committee of Jiangmen Central Hospital and written informed consent was obtained from the participants. A total of 26 patients with NPC, 19 patients with chronic head and neck complaints/nasopharyngitis, and 10 healthy donors were recruited into this study. The diagnoses were histologically confirmed by experienced pathologists. Clinical staging was defined with 2 patients at early stages (I/II) and 24 patients at late stages (III/IV) according to the 8th edition UICC 2017 criteria. All the nasopharyngeal biopsies and swabs were taken from the Department of Otolaryngology, Affiliated Jiangmen Hospital of Sun Yat-sen University.

### 2.2. DNA Preparation and Bisulfite Conversion

Genomic DNA was isolated from nasopharyngeal tissues and swabs using a magnetic bead-based DNA extraction kit (GenePhar Technologies Inc., China). The extracted DNA was treated with bisulfite using a GS DNA methylation kit (GeneShine Biotechnology, China) according to the manufacturer's protocol.

### 2.3. Quantitative Methylation-Sensitive PCR (qMS-PCR)

The bisulfite-modified DNA was immediately subjected to a qMS-PCR assay. The thermal cycling conditions were as follows: 1 cycle at 94°C for 2 min, followed by 50 cycles at 93°C for 30 s, 56°C for 60 s, and 65°C for 30 s. The house keeping gene beta *actin* (*ACTB*) was applied as an internal reference. The quantification cycle (Cq) values were measured at a fixed fluorescence threshold. Samples with a Cq value < 40 as well as the delta Cq (*Septin* 9 − *ACTB*) < 7 were considered to represent a positive test result. Duplicate tests were performed for each sample and an average delta Cq was calculated. *Septin 9* methylation scores were calculated using the following formula: 2^[Cq(ACTB) − Cq(Septin 9)]^ × 100 [14]. All analyses were performed on an ABI 7500 Real-Time PCR System (Applied Biosystems). The methylation site that was queried in the present study mapped upstream to 225-305 bp of the exon 1 of the *Septin 9* transcript isoform v2 (GenBank access no. NM_001113493.1). The relevant sequences for qMS-PCR are shown in Table 1.

### 2.4. Quantitative Reverse Transcription PCR (qRT-PCR)

Nasopharyngeal tissues were stored in RNAStore (Beijing Cowin Bioscience Co., Ltd). Total RNA was extracted with RNAiso Plus (Takara Biomedical Technology Co., Ltd, Beijing) according to the manufacturer's instructions. Subsequently, 1 *μ*g of RNA was pretreated with DNase I and reversely transcripted to first-strand cDNA using Oligo (dT)_20_VN primers and HiScript II One Step RT-PCR Kit (Vazyme Biotech Co., Ltd, Nanjing). Primer set for *Septin 9* amplification was designed to span one intron (1156 bp). The PCR reaction consisted of cDNA template, forward/reverse primers, TB Green *Premix Ex Taq* (Tli RNaseH Plus), and ROX Reference Dye II, in a total volume of 20 *μ*L. The cycling conditions were 95°C for 5 min, followed by 40 cycles of 95°C for 20 s, and 60°C for 1 min. Glyceraldehyde 3-phosphate dehydrogenase (GADPH) was used as the internal control (Table 2). The relative expression level of *Septin 9* mRNA was calculated using the 2^−ΔCt^ × 100 method. All assays were run on an ABI 7500 Fast Real-Time PCR system (Applied Biosystems), and the experiments were performed in triplicate.

### 2.5. Statistical Analysis

Statistical analyses were conducted using GraphPad Prism 5.0 (GraphPad Software Inc., San Diego, CA), and all analyses were two-sided. A chi-squared test was used to analyze the differences in methylation rate of *Septin 9* among all the groups. Kruskal-Wallis test or Mann–Whitney *U* test was used to calculate the differences in methylation score of *Septin 9* among different groups. Pearson correlation was used to analyze the relationship between *Septin 9* methylation and expression at mRNA level in nasopharyngeal tissues. A *p* value of <0.05 was considered statistically significant.

## 3. Results

### 3.1. High-Frequency Methylation and Decreased mRNA Expression of Septin 9 in NPC

**Figure 1:**
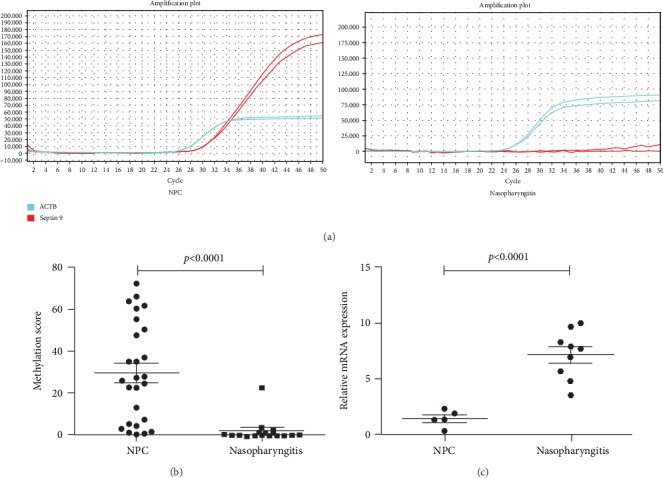
Frequent methylation and downregulation of *Septin 9* in NPC. (a) Amplification curves of *Septin 9* in nasopharyngeal tissues of patients with NPC and nasopharyngitis by qMS-PCR. (b) Comparison of *Septin 9* methylation levels in NPC and nasopharyngitis biopsies. (c) Relative expression of *Septin 9* in NPC and chronic nasopharyngitis. The expression level of *Septin 9* mRNA was determined using qRT-PCR and normalized to the housekeeping gene (GAPDH).

To evaluate the diagnostic value of *Septin 9* for detecting NPC, we used a qMS-PCR assay to examine its methylation status in tissue samples from 25 patients with NPC and 16 subjects with chronic nasopharyngitis. Representative amplification curves of *Septin 9* are shown in Figure 1(a). Methylated *Septin 9* was detected in 23 (92%) NPC cases, including early and late stages NPC. Whereas it was found in 4 (25%) nasopharyngitis controls, showing a marked difference in methylation frequency of *Septin 9* between NPC and non-NPC nasopharyngeal biopsies (*p* < 0.05). To quantitatively compare its methylation level between the two groups, we further calculated their methylation scores of *Septin 9*. As shown in Figure 1(b), a significantly higher methylation level of *Septin 9* was identified in NPC compared with nasopharyngitis control (*p* < 0.0001) using the Mann–Whitney *U* test.

Furthermore, we investigated the mRNA levels of *Septin 9* between NPC and chronic nasopharyngitis by qRT-PCR. From Figure 1(c), we observed a significantly lower *Septin 9* mRNA level in NPC biopsies than that in nasopharyngitis controls (*p* < 0.0001). To establish the correlation between *Septin 9* methylation and expression in nasopharyngeal tissues, methylation score and relative expression of *Septin 9* mRNA were used to perform correlation analysis. For NPC, the Pearson's *r* was −0.498 (*p* > 0.05) and −0.753 (*p* > 0.05) was for nasopharyngitis subjects.

### 3.2. Comparison of Septin 9 Methylation in Nasopharyngeal Swabs and Paired Tissues

**Figure 2:**
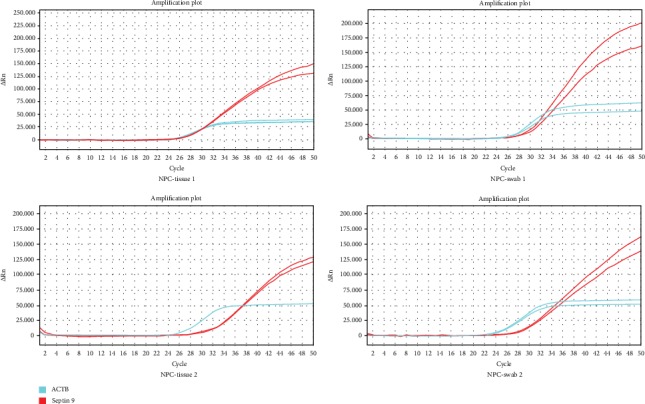
Representative images of amplification curves for analyzing the methylation status of *Septin 9* in nasal swabs and paired tissues of two NPC cases.

Because the nasal swab is a minimally invasive sampling method, we then evaluated its clinical utility for identifying NPC. Nasal swabs and paired tissues from 21 patients with NPC were used to run a qMS-PCR assay (Figure 2). We identified *Septin 9* methylation in 90.5% (19/21) of NPC biopsies and 71.4% (15/21) of corresponding swabs, revealing a concordance rate of 79% between the two sample types (*p* = 0.61). Methylation was not found in swabs whose paired biopsies (2 cases) were *Septin 9*-unmethylated. Our results confirmed that nasal swab could be used to assist the detection of NPC.

### 3.3. Septin 9 Methylation in Nasopharyngeal Swabs with Differing Severity of Nasal Lesions

Since nasal swab is a potential alternative tool for detecting of NPC, we then compared the methylation status of *Septin 9* in nasopharyngeal swabs among three groups with differing severity of nasal lesions. Hypermethylated *Septin 9* was determined in 16 of 22 (72.7%) NPC, whereas it was found in 2 of 19 (10.5%) nasopharyngitis, but not in any of the healthy controls (Table 3), showing a significant difference in methylation frequency between NPC and non-NPC (*p* < 0.01). However, *Septin 9* methylation rates were not related to tumor T classification (T1-2 vs. T3-4) (*p* = 0.78). As mentioned above, 21 nasal swabs were taken from 21 patients with NPC (one swab for each person). In this experiment, we used the same swab samples, plus one NPC case only with nasal swab.

**Figure 3:**
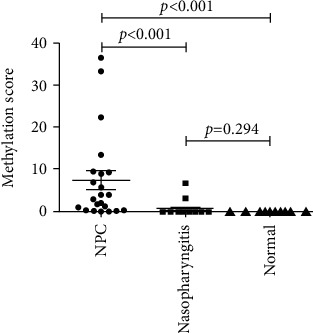
Scatter plot of *Septin 9* methylation scores in nasopharyngeal swabs. Transverse bars represent the mean value and the 95% confidence interval and vertical bars represent the standard deviation, respectively. Comparison of *Septin 9* methylation levels in nasopharyngeal swabs of 22 patients with NPC, 19 subjects with nasopharyngitis, and 10 healthy controls.

Moreover, we compared the methylation score of *Septin 9* in nasal swabs of NPC, chronic nasopharyngitis, and healthy control. The Kruskal-Wallis statistic revealed a significant statistical difference among the three groups (*p* < 0.0001). Furthermore, a significantly higher methylation level of *Septin 9* was identified in NPC compared with non-NPC controls (NPC vs. nasopharyngitis, *p* < 0.001; NPC vs. normal, *p* < 0.001) using Dunn's multiple comparison test (Figure 3). However, no significant difference of it was observed between nasopharyngitis and normal control (*p* > 0.05).

### 3.4. Diagnostic Capacity of Septin 9 Methylation in NPC vs. Non-NPC Controls

**Figure 4:**
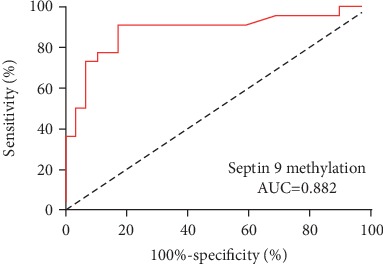
The diagnostic power of *Septin 9* methylation. ROC curve analysis showed an AUC of 0.882 of *Septin 9* methylation tests in detecting NPC.

To evaluate the clinical performance of *Septin 9* methylation for diagnosing NPC, we constructed the receiver operating characteristic (ROC) curves and calculated the area under the curve (AUC). The larger the area under the ROC curve presents the higher the diagnostic power. As shown in Figure 4, an AUC of *Septin 9* methylation was up to 0.882 (95% confidence interval = 0.777–0.986, *p* < 0.0001). Our data indicated that testing for *Septin 9* methylation provides a potential method in differentiating NPC from non-NPC subjects.

## 4. Discussion

Although each cell type has a unique DNA methylation pattern, aberrant epigenetic alterations have been regarded as a common hallmark of human cancers. Prior studies demonstrated that *Septin 9* was hypermethylated in a broad spectrum of tumors such as colorectal cancer, breast cancer, and cervical cancer. Since the *Septin 9* gene has not been analyzed in NPC before, this study for the first time aimed to investigate its potential as a new biomarker for the early detection of NPC. Herein, we identified *Septin 9* frequently methylated in 92% of NPC tissues, which has a higher methylation frequency compared with other genes reported being hypermethylated in NPC [24]. The *Septin 9* methylation level of NPC group was also significantly higher than that of the non-NPC controls (*p* < 0.001). Compared with chronic nasopharyngitis, NPC subjects had a reduced mRNA level of *Septin 9* (*p* < 0.0001), indicating a close association between DNA methylation and transcriptional silencing of *Septin 9* in NPC. Similar observation has been also documented in colorectal cancer [25]. However, we failed to observe a significant correlation between *Septin 9* methylation and mRNA level in individuals (*p* > 0.05) in this investigation, although methylation negatively regulating mRNA expression of *Septin 9* is mentioned above. Nasopharyngeal biopsy usually provides limited amount of tissue volume, which will result in varying percentages of diseased cells, especially cancer cells, in the specimens. This may contribute to interindividual variations of *Septin 9* methylation and expression among the subjects. Moreover, ROC curve analysis showed an AUC of 0.882 of *Septin 9* methylation tests to discriminate NPC from non-NPC subjects, indicating that epigenetic silencing of *Septin 9* may function importantly in the carcinogenesis of NPC.

Further, we found that methylation status of *Septin 9* in nasal swabs of recruited subjects was correlated well with that in paired NPC tissues, which is in agreement with a previous study [26]. As a surface mucosal tumor, NPC cells are accessible by nasal swabs. Moreover, cancer cells can be easily released from tumor mass and obtained when cell-cell adhesion is destroyed that frequently occurred in malignant tumors. Therefore, analysis of *Septin 9* methylation in nasal swabs may provide a minimally invasive way in assisting diagnosing of NPC or monitoring recurrence after treatment. In addition, nasal swabs is possible to become a population screening tool for NPC due to its simplicity and convenience, in particular, in the communities of Guangdong province. In case a positive test result is determined, otorhinolaryngologists may perform additional examinations to confirm it.

In patients with chronic head and neck complaints/nasopharyngitis, two of 19 cases were found carrying *Septin 9* methylation in nasal swabs. To double check the results, resampling was carried out and subjected to a qMS-PCR assay again. Strong amplification signals of methylated *Septin 9* were present in these samples. Although current pathological examination did not find cancer cells, they may have an increased risk of NPC. Aberrant DNA methylation is one of the earliest event in the tumorgenic process and often present in the precursor lesions of human cancers [27]. Thus, we speculated that hypermethylation of *Septin 9* may occur in the early stage of human cancers such as cervical cancer and NPC that contribute directly to cancer onset and progression. In addition, Jiangmen city, located in Guangdong province, is one of the top 10 NPC incidence and mortality areas in China [28]. Methylation changes preceding malignancy have been described in many cancers. As demonstrated in a previous study, hypermethylation of *FAM19A4*/*mir124-2* was found in cervical scrapes of HPV-positive women with normal cytology at the time of sampling who developed cervical cancer five and ten years later [29]. Eads et al. found frequent methylation of specific genes present in nondysplastic Barrett's oesophagus and associated adenocarcinoma [30]. Other reports revealed *p16* hypermethylation in gastric mucosa of five patients with gastric dysplasia who subsequently developed gastric carcinomas five years later [31]. Similarly, *p16* methylation was detectable in oral epithelial dysplasia lesions of patients up to ~45 months before clinical and pathological diagnosis of oral squamous cell carcinomas [32]. Therefore, methylation imbalance is not just a consequence of malignant transformation. It might serve as a predictive marker in normal appearing samples from “at risk” individuals. This is especially important and helpful in clinical practice if patients with early symptoms suspected of having NPC or NPC high-risk population can be picked out using a single methylation marker. Taken together, we predict the patients with chronic head and neck complaints carrying methylated *Septin 9* at high risk of developing NPC and we will follow up these subjects for a definitive diagnosis.

Our pilot study has several limitations. First, the project shows the possibility of *Septin 9* methylation-based approach to aid NPC diagnosis with relatively small sample size. Larger cohorts are needed to confirm its diagnostic accuracy. Second, the present study is lacking in follow-up examinations. We will collect longitudinal clinical samples to conduct a prospective study to evaluate the predictive ability of *Septin 9* methylation in the local high-risk subpopulation.

## 5. Conclusions

Our data demonstrated that *Septin 9* methylation was frequently present in NPC. Its detection in nasopharyngeal swabs may provide a minimally invasive and informative means for early NPC detection.

## Figures and Tables

**Table 1:** Primers and probes used for qMS-PCR.

Primer/probe	Sequence (5′-3′)
*Septin 9*-F	TTTAGTTAGCGCGTAGGGTTC
*Septin 9*-R	AACTAATAAACAACGAATCGCG
*Septin 9*-probe	FAM-ACGCCCCCGACGAAACC- BHQ1
*ACTB*-F	ATAATAAAAAGGAGGTTGGAT
*ACTB*-R	CTCCCRCAAAACAACCAC
*ACTB*-probe	VIC-CCACCTTACCCTAAACACTACAAC-BHQ1

qMS-PCR: quantitative methylation-sensitive PCR.

**Table 2:** Primer sets designed for qRT-PCR.

Primer set	Sequence (5′-3′)	Amplicon length (bp)
*Septin 9*-F	CTCATCAGGACGCACATGCAG	147
*Septin 9*-R	CGTCTACATCTCCGGGGCTT	
*GAPDH*-F	CAAGCTCATTTCCTGGTATGACA	189
*GAPDH*-R	GGGAGATTCAGTGTGGTGGG	

qRT-PCR: quantitative reverse transcription PCR.

**Table 3:** Identification of *Septin 9* methylation in nasopharyngeal swabs.

Subjects	Total cases	No. of methylated *Septin 9*	Methylation rates (%)	*p* value
NPC	22	16	72.7 (16/22)	0.009/0.012^a^
T1-2	12	8	66.7 (8/12)	0.78
T3-4	10	8	80 (8/10)	
Nasopharyngitis	19	2	10.5 (2/19)	0.31
Healthy control	10	0	0 (0/10)	

^a^ *p* = 0.009 for NPC vs. nasopharyngitis; *p* = 0.012 for NPC vs. healthy control.

## Data Availability

Answer: Yes. Comment: The data used to support the findings of this study are available from the corresponding author upon request.
